# Herpes simplex virus type 1 DNA is associated with changes in MRI outcomes in an AD‐dependent pattern

**DOI:** 10.1002/alz70855_107340

**Published:** 2025-12-25

**Authors:** Ali Gulhan, Marlene Tejeda, Caitlin Newman, John J. Farrell, Congcong Zhu, Lee Wetzler, Kathryn L. Lunetta, William S Bush, Eden R. Martin, Li‐San Wang, Gerald D. Schellenberg, Margaret Pericak‐Vance, Jonathan L Haines, Lindsay A. Farrer, Richard Sherva, The Framingham Heart Study

**Affiliations:** ^1^ Boston University School of Medicine, Boston, MA, USA; ^2^ Boston University, Boston, MA, USA; ^3^ Biomedical Genetics, Department of Medicine, Boston University Medical School, Boston, MA, USA; ^4^ Framingham Heart Study, Boston University Chobanian & Avedisian School of Medicine, Boston, MA, USA; ^5^ Framingham Heart Study, Framingham, MA, USA; ^6^ Department of Population and Quantitative Health Sciences, Case Western Reserve University School of Medicine, Cleveland, OH, USA; ^7^ Dr. John T. Macdonald Foundation Department of Human Genetics, University of Miami Miller School of Medicine, Miami, FL, USA; ^8^ University of Pennsylvania, Philadelphia, PA, USA; ^9^ Penn Neurodegeneration Genomics Center, Department of Pathology and Laboratory Medicine, University of Pennsylvania Perelman School of Medicine, Philadelphia, PA, USA; ^10^ The John P. Hussman Institute for Human Genomics, University of Miami, Miami, FL, USA; ^11^ Department of Population & Quantitative Health Sciences, School of Medicine, Case Western Reserve University, Cleveland, OH, USA; ^12^ Department of Neurology and Ophthalmology, Boston University Chobanian & Avedisian School of Medicine, Boston, MA, USA; ^13^ Boston University Chobanian & Avedisian School of Medicine, Boston, MA, USA; ^14^ Boston University Alzheimer's Disease Research Center, Boston University Chobanian & Avedisian School of Medicine, Boston, MA, USA; ^15^ Department of Biostatistics, Boston University School of Public Health, Boston, MA, USA

## Abstract

**Background:**

Herpes Simplex Virus Type 1 (HSV‐1) has been implicated in the pathogenesis of Alzheimer's Disease (AD) in multiple studies. This cross‐sectional study investigates the association between HSV‐1 and AD using magnetic resonance imaging (MRI) and whole‐genome sequencing data from 473 participants (35 AD cases, 438 controls) in the Framingham Heart Study (FHS) and 841 participants (231 AD cases, 87 MCI cases, 515 controls) from the Alzheimer's Disease Sequencing Project (ADSP) cohorts.

**Methods:**

DNA sequence reads that did not map to the human genome were aligned to HSV‐1 viral reference sequences to determine viral presence. Associations between HSV‐1 viral presence and alterations in brain volume and cortical thickness were assessed in the two study cohorts. The association between HSV‐1 DNA and log normalized MRI outcomes were tested with linear regression models including AD diagnosis, age, sex, and APOE genotype as covariates. Where multiple MRI measures were available, the most recent was used. The main effect of HSV‐1 and the interaction of AD/ mild cognitive impairment (MCI) x HSV‐1 DNA was analysed. The FHS and ADSP results within regions were combined via meta‐analysis.

**Results:**

In the main effect, HSV‐1 DNA is associated with significant differences (*p* < 0.05) across 19 regions, with 12 regions showing increased volumes and 7 region demonstrating decreases. HSV‐1 showed significant interaction with AD/MCI in six regions. The joint effect of HSV‐1 DNA was associated with an increase in surface holes (β=0.39, *p* = 4.5x10^‐3^), and accelerated thinning in the accumbens (β=‐0.1, *p* = 1.6x10^‐2^), inferior parietal (β=‐0.048, *p* = 9.2x10^‐3^), lateral occipital (β=‐0.043, *p* = 1.7x10^‐2^), middle temporal (β=‐0.012, *p* = 3.2x10^‐2^), and reductions in temporal pole volume (β=‐0.069, *p* = 3.3x10^‐2^). No associations between HSV‐1 and MRI measures were found in cognitively normal individuals when considering the joint effect of HSV‐1.

**Conclusion:**

These findings suggest that HSV‐1 may exacerbate cerebral atrophy associated with Alzheimer's Disease. However, further studies are needed to confirm these results and elucidate underlying mechanisms.

